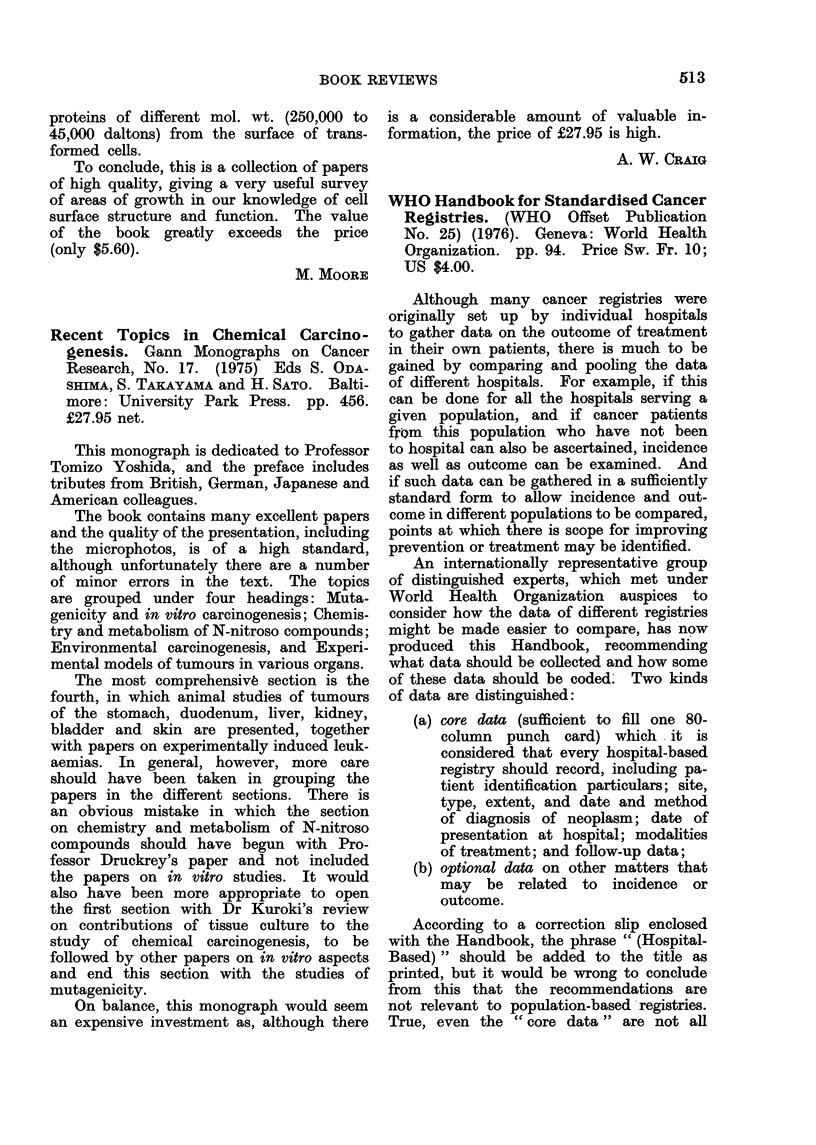# Recent Topics in Chemical Carcinogenesis

**Published:** 1977-04

**Authors:** A. W. Craig


**Recent Topics in Chemical Carcinogenesis.** Gann Monographs on Cancer Research, No. 17. (1975) Eds S. ODASHIMA, S. TAKAYAMA and H. SATO. Baltimore: University Park Press. pp. 456. £27.95 net.

This monograph is dedicated to Professor Tomizo Yoshida, and the preface includes tributes from British, German, Japanese and American colleagues.

The book contains many excellent papers and the quality of the presentation, including the microphotos, is of a high standard, although unfortunately there are a number of minor errors in the text. The topics are grouped under four headings: Mutagenicity and *in vitro* carcinogenesis; Chemistry and metabolism of N-nitroso compounds; Environmental carcinogenesis, and Experimental models of tumours in various organs.

The most comprehensive section is the fourth, in which animal studies of tumours of the stomach, duodenum, liver, kidney, bladder and skin are presented, together with papers on experimentally induced leukaemias. In general, however, more care should have been taken in grouping the papers in the different sections. There is an obvious mistake in which the section on chemistry and metabolism of N-nitroso compounds should have begun with Professor Druckrey's paper and not included the papers on *in vitro* studies. It would also have been more appropriate to open the first section with Dr Kuroki's review on contributions of tissue culture to the study of chemical carcinogenesis, to be followed by other papers on *in vitro* aspects and end this section with the studies of mutagenicity.

On balance, this monograph would seem an expensive investment as, although there is a considerable amount of valuable information, the price of £27.95 is high.

A. W. CRAIG